# The relationship between loneliness, depressive symptoms, and self-perception of ageing among older adults

**DOI:** 10.1186/s12877-026-07921-w

**Published:** 2026-06-29

**Authors:** Beata Boratyn, Małgorzata Dziechciaz, Dorota Talarska

**Affiliations:** 1 Faculty of Health Sciences, State Academy of Applied Sciences named after Bronisław Markiewicz in Jarosław, Czarnieckiego Street, Jarosław, 37-500 Poland; 2 Department of Preventive Health, Poznan University of Medical Sciences, Poznań, Poland

**Keywords:** Older adults, Attitudes towards ageing, Loneliness, Depressive symptoms

## Abstract

**Background:**

The proportion of older adults is increasing in nearly all societies worldwide. Understanding how older adults perceive their own ageing has therefore become increasingly important. The aim of this study was to examine the relationship between loneliness, depressive symptoms, and self-perception of ageing among older adults.

**Methods:**

The study included 439 adults aged 60 years and older. The following research instruments were used: the Polish version of the Attitudes to Ageing Questionnaire (AAQ-24-PL), the 15-item Geriatric Depression Scale (GDS-15) according to Yesavage, the De Jong Gierveld Loneliness Scale (DJGLS), and an author-designed interview questionnaire.

**Results:**

The mean overall AAQ score was 75.74 (SD = 11.82). Both overall self-perception of ageing and its individual domains were significantly associated with loneliness (AAQ: *p* < 0.001, OR = 2.59; PL: *p* < 0.001, OR = 2.76; PC: *p* < 0.001, OR = 3.37; PG: *p* < 0.001, OR = 3.59), depressive symptoms (AAQ: *p* < 0.001, OR = 2.07; PL: *p* < 0.001, OR = 2.35; PC: *p* < 0.001, OR = 4.92; PG: *p* < 0.001, OR = 3.12), social network characteristics (AAQ: *p* = 0.006, OR = 1.72; PL: *p* = 0.002, OR = 1.84; PC: *p* = 0.001, OR = 2.26; PG: *p* = 0.005, OR = 1.75), self-rated health (AAQ: *p* < 0.001, OR = 2.07; PL: *p* < 0.001, OR = 2.20; PC: *p* < 0.001, OR = 2.73; PG: *p* = 0.021, OR = 1.60), and age (AAQ: *p* = 0.019, OR = 1.60; PL: *p* = 0.008, OR = 1.69; PC: *p* = 0.004, OR = 2.07; PG: *p* = 0.026, OR = 1.57). Active leisure activities (PC: OR = 0.46; PG: OR = 0.64) and higher educational attainment (PC: OR = 0.29; PG: OR = 0.48) were associated with a more positive self-perception of ageing.

**Conclusions:**

Respondents demonstrated positive attitudes towards their own ageing in the domains of physical change and psychological growth, whereas more negative attitudes were observed in the psychosocial loss domain. Loneliness and depressive symptoms were significantly associated with a more negative self-perception of ageing among older adults. A more negative self-perception of ageing was also more frequently observed among respondents with a poorly developed social network, poorer self-rated health, and older age. Conversely, active leisure engagement and higher educational attainment were associated with a more positive self-perception of ageing.

## Introduction

Increasing life expectancy has led ageing policies to focus increasingly on the concept of healthy ageing, understood as maintaining the highest possible level of functioning, promoting the active participation of older adults in social life, and accepting the natural changes associated with ageing [1]. Attitudes towards one’s own ageing reflect relatively stable patterns of thinking, feeling, and behaviour related to the ageing process. They encompass beliefs about physical and psychological changes, emotional responses to the passage of time, and readiness to adapt to age-related changes and undertake new social roles [2, 3]. These attitudes may influence how older adults cope with and experience the ageing process [4].

Attitudes towards ageing are not static but develop throughout the life course and may change over time. They are shaped by sociocultural beliefs, historical context, traditions, and religion [5]. At the individual level, demographic characteristics such as age [6], sex, marital status [7], educational attainment [8, 9], socioeconomic status, and health status [6] also play an important role. Psychological factors, including self-esteem [10] and depression [11], as well as aspects of social functioning such as interpersonal relationships, frequency of contact with significant others, social network structure, social support, and social participation, have also been shown to influence attitudes towards ageing [9, 12].

Attitudes towards one’s own ageing are closely linked to quality of life in older adults. Individuals with positive attitudes towards ageing tend to perceive later life as an opportunity for continued personal development and self-fulfilment while accepting the limitations associated with this stage of life [13]. In contrast, those with more negative attitudes are more likely to perceive ageing as a period characterised by loneliness, social exclusion, and loss of independence [4]. Negative attitudes towards ageing may also contribute to social withdrawal.

In this context, loneliness becomes particularly important. Although loneliness occurs across all age groups, older adults are especially vulnerable because of increasing health problems, reduced mobility, widowhood, and the loss of social roles and social networks associated with retirement [14]. The reported prevalence of loneliness among older adults ranges from approximately 25% to 70%, depending on the study population and the assessment method used. In a meta-analysis, Salari et al. estimated the global prevalence of loneliness among older adults to be 27.6% [15]. Loneliness is defined as a subjective feeling arising from a discrepancy between desired and actual social relationships [16]. It reflects the perception that social contacts are insufficient or unsatisfactory and is often accompanied by feelings of alienation. Loneliness is closely related to social isolation, which is manifested by a limited number of social contacts or their complete absence. When persistent, loneliness may adversely affect both health and well-being, including contributing to the development of depressive symptoms [17].

The relationship between loneliness and depressive symptoms has been demonstrated in several studies. For example, Theeke (2009) compared older adults experiencing loneliness with those who did not report such feelings and found that lonely individuals were nearly twice as likely to experience depressive symptoms [18]. Similar associations have also been confirmed in other longitudinal studies [19].

These findings suggest that attitudes towards ageing, loneliness, and depressive symptoms are interrelated. A negative self-perception of ageing may be associated with social withdrawal and more severe depressive symptoms. Conversely, loneliness and depressive symptoms may reinforce negative perceptions of ageing, suggesting a potentially bidirectional relationship between these factors. These associations may be interpreted in the light of Levy’s Stereotype Embodiment Theory, which proposes that internalised age stereotypes influence psychological, social, and physical functioning throughout later life [20].

Although the number of studies examining loneliness, depression, and attitudes towards ageing has increased, relatively few have investigated these variables simultaneously. The present study extends the existing evidence by simultaneously examining loneliness, depressive symptoms, and the multidimensional construct of self-perception of ageing using the three domains of the AAQ-24-PL. 

## Methods

### Aim

The aim of this study was to examine the relationship between loneliness, depressive symptoms, and self-perception of ageing among older adults. The study was designed as a cross-sectional observational study. In addition, an exploratory cluster analysis was performed to identify groups of respondents presenting similar patterns of attitudes towards ageing.

### Study setting

The study was conducted between February and December 2024. Before enrolment, all participants were informed about the study objectives and their right to withdraw at any stage without providing a reason. Written informed consent was obtained from all participants. Prior to the main stage of the study, cognitive functioning was assessed using the Abbreviated Mental Test Score (AMTS). Only individuals scoring between 7 and 10 points were eligible to participate.

### Study participants

The inclusion criteria were: age ≥ 60 years, ability to move independently, and normal cognitive functioning defined as an AMTS score of ≥ 7 points.

The exclusion criteria were: an AMTS score < 7 points and mobility limitations preventing independent completion of the interview questionnaire.

Mobility was assessed based on participants’ self-reported ability to participate in the study independently without assistance from another person.

A non-probability convenience sampling approach was used. Participants were recruited from Universities of the Third Age (UTA), Senior Day Care Clubs (SDCC), and primary healthcare (PHC) outpatient clinics. It should be noted that these settings were not mutually exclusive. Some respondents attending primary healthcare clinics also participated in activities organised by Universities of the Third Age or Senior Day Care Clubs. Therefore, participation in these settings was analysed as an indicator of social activity rather than as separate recruitment groups.

The minimum required sample size was estimated at 380 participants according to the recommendations of Tabachnick and Fidell. After accounting for the anticipated effect size and statistical power, the minimum sample size was estimated at 387 participants. Ultimately, data were collected from 439 respondents, which reduced the maximum estimation error.

### Research instruments

Data were collected using face-to-face interviews. The following research instruments were used:

- An author-designed interview questionnaire, including sociodemographic characteristics, family relationships, leisure activities, and self-rated health.
- The Attitudes to Ageing Questionnaire (AAQ-24-PL) [21]. The AAQ consists of 24 items grouped into three domains: Psychosocial Loss (PL), Physical Change (PC), and Psychological Growth (PG). Example items include: *“Old age is a time of loneliness”* (PL), *“Physical exercise is important at any age”* (PC), and *“Wisdom comes with age”* (PG). Each item is rated on a five-point Likert scale ranging from *strongly agree* to *strongly disagree*. Scores for each domain range from 8 to 40 points, while the total score ranges from 24 to 120 points. Higher scores indicate more positive attitudes towards one’s own ageing. Reported internal consistency (Cronbach’s alpha) is 0.84 for Psychosocial Loss, 0.78 for Physical Change, 0.73 for Psychological Growth, and 0.89 for the overall scale [21]. In the present study, Cronbach’s alpha was 0.78 for the total scale and ranged from 0.57 to 0.80 across the individual domains.
- The 15-item Geriatric Depression Scale (GDS-15) developed by Yesavage [22]. The GDS-15 is a widely used and validated screening instrument for depressive symptoms in older adults. It consists of 15 yes/no questions. An example item is: *“Do you feel that your life is empty?”* Total scores range from 0 to 15 points, with higher scores indicating more severe depressive symptoms [22]. In the present study, Cronbach’s alpha for the GDS-15 was 0.79.
- The De Jong Gierveld Loneliness Scale (DJGLS) [23]. Example items include: *“I experience a general sense of emptiness”* and *“There are plenty of people I can rely on when I have problems.”* The Polish version of the scale was validated by Grygiel et al. in 2012. The reliability and validity of the Polish version are high (Cronbach’s alpha = 0.89) [24]. In the present study, Cronbach’s alpha for the total scale was 0.86.
- The Abbreviated Mental Test Score (AMTS) was used as a screening instrument to assess cognitive functioning in older adults. Individuals scoring fewer than 7 points were excluded because cognitive impairment could interfere with their ability to provide reliable responses [25].

### Statistical analysis

Results are presented using descriptive statistics (mean with 95% confidence interval and standard deviation). Cronbach’s alpha coefficients and Pearson’s correlation coefficients were calculated for the standardised instruments. Associations between categorical variables were assessed using the chi-square test of independence (χ²).

K-means cluster analysis was performed separately for the overall AAQ score and for each of the three AAQ domains (PL, PC, and PG) to identify groups of respondents presenting similar patterns of attitudes towards ageing. For each analysis, two- and three-cluster solutions were compared. In all analyses, the two-cluster solution was selected because it provided greater interpretability and enabled the identification of two clearly distinct groups representing positive and negative attitudes towards ageing. The quality of the cluster solutions was assessed in terms of interpretability. Formal assessment of cluster stability was not performed. Differences between the identified clusters were assessed using one-way analysis of variance (ANOVA) for continuous variables and the chi-square test of independence (χ²) for categorical variables.

Based on the obtained cluster solutions, empirical cut-off points corresponding to the boundaries between the identified clusters were established: 78 points for the overall AAQ score and 23, 23, and 28 points for the PL, PC, and PG domains, respectively. Associations between cluster membership and selected sociodemographic, health-related, and psychosocial variables were subsequently examined.

Variables included in the logistic regression analyses, including social network, family relationships, leisure activities, financial situation, and educational attainment, were recoded into dichotomous categories based on empirical groupings obtained using hierarchical cluster analysis with Ward’s method. Cluster membership was coded as a dichotomous outcome variable and used in logistic regression analyses to estimate odds ratios (ORs). Separate logistic regression models were fitted for each independent variable. The reported odds ratios were derived from univariable logistic regression models and represent the associations between individual variables and the likelihood of negative attitudes towards ageing. Statistical significance was set at *p* < 0.05. All analyses were performed using IBM SPSS Statistics version 22.0.

## Results

A total of 439 adults aged 60 years and older were included in the study. The mean age of the participants was 72.1 years. Women accounted for 71.1% of the study population (*N* = 312), whereas men represented 28.9% (*N* = 127). The largest proportion of respondents was aged 71–80 years (46.7%; *N* = 205). Widowed participants constituted the largest marital status group (44.2%), followed by married participants (39.9%). Regarding educational attainment, the largest groups had secondary education (38.0%; *N* = 167) and vocational education (26.7%; *N* = 117). Financial situation was most frequently rated as good (52.6%; *N* = 231), while 33.3% of respondents rated it as average. The respondents most commonly lived alone (37.4%; *N* = 164), with family members (31.4%; *N* = 138), or with a spouse (27.3%; *N* = 120). Family relationships were most frequently rated as good (54.7%; *N* = 240), with nearly one-third of respondents rating them as very good (31.2%; *N* = 137).

Participants reported maintaining social contacts primarily with family members (85.4%; *N* = 375), followed by acquaintances (58.8%; *N* = 258) and friends (34.6%; *N* = 152). Meetings with significant others most frequently occurred on a daily basis (45.1%; *N* = 195), whereas 11.0% of respondents reported meeting only during holidays or having no contact at all. Watching television was the most common leisure activity (61.0%; *N* = 268). Other leisure activities included spending time with family and friends (36.4%; *N* = 160), participation in Universities of the Third Age (31.2%; *N* = 137), pursuing personal interests (31.2%; *N* = 137), and spending time with grandchildren (28.5%; *N* = 125). The mean self-rated health score was 2.77 (SD = 0.82). Nearly half of the respondents rated their health as neither good nor poor (48.1%; *N* = 211), whereas 33.3% (*N* = 146) rated it as good.

Additional analyses according to participation in UTA and SDCC activities revealed several sociodemographic differences. Participants attending UTA were more likely to be aged 60–70 years (51.8% vs. 30.1%; *p* < 0.001), to be married (46.7% vs. 36.8%; *p* = 0.048), and to report a good or very good financial situation (83.9% vs. 54.3%; *p* < 0.001). They also more frequently rated their family relationships as very good (38.7% vs. 27.8%; *p* = 0.023) and less frequently reported depressive symptoms (24.1% vs. 46.4%). In contrast, participants attending SDCC were more likely to be older than 70 years (88.6% vs. 57.5%; *p* < 0.001), to be unmarried, widowed, or divorced (87.3% vs. 54.2%; *p* < 0.001), and to report daily contact with significant others (60.8% vs. 41.7%; *p* = 0.002). They were also more likely to report a poorer financial situation (48.1% vs. 33.9%; *p* = 0.017).

The sociodemographic characteristics of the study population are presented in Table 1.

**Table 1:** Sociodemographic characteristics of the study population

Sociodemographic variable	*N*	%		*N*	%
Sex			Living arrangement		
Women	312	71.1	Living alone	164	37.4
Men	127	28.9	Living with a spouse	120	27.3
Age			Living with family	138	31.4
60–70 years	162	36.9	Living with unrelated persons	4	0.9
71–80 years	205	46.7	Living with a partner	13	3.0
≥ 81 years	72	16.4			
Marital status			Family relationships		
Single	31	7.1	Very good	137	31.2
Married	175	39.9	Good	240	54.7
Divorced	25	5.7	Neutral	46	10.5
Widowed	194	44.2	Poor	5	1.1
Separated	7	1.6	No contact with family	11	2.5
Informal relationship	7	1.6	Frequency of meetings with significant others		
Education			Daily	198	45.1
Incomplete primary	0	0.0	Once a week	113	25.7
Primary	63	14.4	2–3 times per month	76	17.3
Vocational	117	26.7	Only during holidays	35	8.0
Secondary	167	38.0	No contact	13	3.0
Higher	92	21.0	Other	4	0.9
Financial situation			Self-rated health		
Very good	48	10.9	Very good	17	3.9
Good	231	52.6	Good	146	33.3
Average	146	33.3	Neither good nor poor	211	48.1
Poor	14	3.2	Poor	52	11.8
Leisure activities^*^			Difficult to assess	13	3.0
Spending time with family and friends	160	36.4	Social contacts maintained^*^		
Watching television	268	61.0	Family	375	85.4
Spending time with grandchildren	125	28.5	Acquaintances	258	58.8
Pursuing personal interests	137	31.2	Friends	152	34.6
Participation in the University of the Third Age	137	31.2	Neighbours	200	45.6
Senior Day Care Club	79	18.0	No one	9	2.1
Other	14	3.2	Other	5	1.1

^*^Multiple-response question; therefore, percentages do not total 100%

### Depressive symptoms and loneliness

The prevalence of depressive symptoms was as follows that 60.6% (*N* = 266) of the respondents had no depressive symptoms, whereas 29.8% (*N* = 131) presented moderate depressive symptoms and 9.6% (*N* = 42) presented severe depressive symptoms. The mean GDS-15 score was 4.97 (SD = 3.76). Loneliness was reported by 57.4% (*N* = 252) of the respondents, including moderate loneliness in 43.5% (*N* = 191), severe loneliness in 8.7% (*N* = 38), and very severe loneliness in 5.2% (*N* = 23). The mean score on the De Jong Gierveld Loneliness Scale was 4.08 (SD = 3.47).

### Attitudes towards ageing

The mean overall AAQ score was 75.74 (SD = 11.82). The highest mean score was observed in the Psychological Growth domain (28.10), followed by the Physical Change (25.39) and Psychosocial Loss (22.25) domains (Table 2).

**Table 2:** Mean AAQ scores overall and by domain

Domain	Mean (M)	SD	Min	Max
Psychosocial Loss	22.25	6.70	8	40
Physical Change	25.39	4.84	9	40
Psychological Growth	28.10	4.99	11	40
Overall AAQ score	75.74	11.82	35	117

*M* mean, *SD* standard deviation, *Min* minimum, *Max* maximum

### Cluster analysis

K-means cluster analysis identified two distinct groups of respondents representing positive and negative attitudes towards ageing. Associations between cluster membership and selected sociodemographic and psychosocial variables are presented in Table 3.

**Table 3:** Associations between selected variables and attitudes towards ageing

Variable	Category	Positive attitude *N*	Positive attitude %	Negative attitude *N*	Negative attitude %	χ² test / *p* value
Sex	Women	167	53.5	145	46.5	χ²(1) = 1.091; *p* = 0.296
	Men	61	48.0	66	52.0	
Age	60–70 years	96	59.3	66	40.7	χ²(1) = 5.516; *p* = 0.019
	> 70 years	132	47.7	145	52.3	
Marital status	Married	104	59.4	71	40.6	χ²(1) = 6.544; *p* = 0.011
	Single/informal relationship	124	47.0	140	53.0	
Education	Primary/vocational	91	50.6	89	49.4	χ²(1) = 0.233; *p* = 0.629
	Secondary/higher	137	52.9	122	47.1	
Financial situation	Very good/good	143	51.3	136	48.7	χ²(1) = 0.143; *p* = 0.706
	Average/poor	85	53.1	75	46.9	
Living arrangement	Living alone	74	45.1	90	54.9	χ²(1) = 4.870; *p* = 0.027
	Living with another person	154	56.0	121	44.0	
Family relationship quality	Very good	80	58.4	57	41.6	χ²(1) = 3.327; *p* = 0.068
	Good or poorer	148	49.0	154	51.0	
Social network	Well-developed	112	59.6	76	40.4	χ²(1) = 7.685; *p* = 0.006
	Poorly developed	116	46.2	135	53.8	
Frequency of meetings with significant others	Daily	112	56.6	86	43.4	χ²(1) = 3.097; *p* = 0.079
	Once a week or less	116	48.1	125	51.9	
Leisure activity	Mainly immediate social environment	140	55.3	113	44.7	χ²(1) = 2.765; *p* = 0.096
	University of the Third Age /personal interests	88	47.3	98	52.7	
Self-rated health	Very good/good	103	63.2	60	36.8	χ²(1) = 13.154; *p* < 0.001
	Fair or poor	125	45.3	151	54.7	
Depressive symptoms	No depressive symptoms	157	59.0	109	41.0	χ²(1) = 13.579; *p* < 0.001
	Moderate/severe depressive symptoms	71	41.0	102	59.0	
Loneliness (DJGLS)	Absent	122	65.2	65	34.8	χ²(1) = 23.100; *p* < 0.001
	Present	106	42.1	146	57.9	

χ², chi-square test; *p* < 0.05 was considered statistically significant

### Sociodemographic variables

Statistically significant associations were observed between attitudes towards ageing and age (*p* = 0.019), marital status (*p* = 0.011), and living arrangement (*p* = 0.027). Respondents aged 60–70 years were more likely to present positive attitudes towards ageing (59.3%) than those aged over 70 years, among whom negative attitudes predominated (52.3%). A significant association was also found for marital status. Married respondents more frequently demonstrated positive attitudes towards ageing (59.4%), whereas 53.0% of unmarried respondents or those in non-marital relationships presented negative attitudes. Similarly, respondents living with another person more often reported positive attitudes (56.0%), whereas negative attitudes predominated among those living alone (54.9%). No statistically significant associations were found between attitudes towards ageing and sex (*p* = 0.296), educational attainment (*p* = 0.629), or financial situation (*p* = 0.706).

### Social functioning variables

Regarding social functioning, a well-developed social network was significantly associated with positive attitudes towards ageing (59.6%) compared with respondents with a poorly developed social network (*p* = 0.006). The strongest association with negative attitudes towards ageing was observed for loneliness. Respondents who did not report loneliness were more likely to present positive attitudes (65.2%), whereas those experiencing loneliness more frequently demonstrated negative attitudes (57.9%; *p* < 0.001). No statistically significant associations were observed between attitudes towards ageing and family relationship quality (*p* = 0.068), frequency of meetings with significant others (*p* = 0.079), or preferred leisure activities (*p* = 0.096).

### Health-related variables

Respondents who rated their health as very good or good significantly more often demonstrated positive attitudes towards ageing (63.2%) than those rating their health as fair or poor (*p* < 0.001), among whom 54.7% presented negative attitudes towards ageing. Likewise, respondents with moderate or severe depressive symptoms significantly more frequently demonstrated negative attitudes towards ageing (59.0%; *p* < 0.001).

Odds ratios (ORs) were calculated to estimate the likelihood of negative self-perception of ageing in the domains of Psychosocial Loss, Physical Change, Psychological Growth, and the overall AAQ score in relation to the variables described above (OR with 95% confidence intervals). OR values greater than 1 indicated a higher likelihood of negative attitudes towards ageing, whereas OR values below 1 indicated a lower likelihood of such attitudes (Table 4).

**Table 4:** Odds ratios for negative self-perception of ageing according to selected variables

Variable	PL OR (95% CI); *p*	PC OR (95% CI); *p*	PG OR (95% CI); *p*	AAQ OR (95% CI); *p*
Depressive symptoms	2.35 (1.58–3.49); *p* < 0.001	4.92 (3.26–7.43); *p* < 0.001	3.12 (2.10–4.65); *p* < 0.001	2.07 (1.40–3.05); *p* < 0.001
Loneliness	2.76 (1.87–4.08); *p* < 0.001	3.37 (2.24–5.07); *p* < 0.001	3.59 (2.39–5.41); *p* < 0.001	2.59 (1.75–3.82); *p* < 0.001
Self-rated health	2.20 (1.48–3.26); *p* < 0.001	2.73 (1.81–4.14); *p* < 0.001	1.60 (1.07–2.37); *p* = 0.021	2.07 (1.39–3.08); *p* < 0.001
Social network	1.84 (1.25–2.70); *p* = 0.002	2.26 (1.52–3.35); *p* < 0.001	1.75 (1.19–2.58); *p* = 0.005	1.72 (1.17–2.51); *p* = 0.006
Age	1.69 (1.14–2.50); *p* = 0.008	2.07 (1.38–3.10); *p* < 0.001	1.57 (1.06–2.33); *p* = 0.026	1.60 (1.08–2.37); *p* = 0.019
Family relationship quality	1.67 (1.11–2.51); *p* = 0.013	3.37 (2.14–5.29); *p* < 0.001	2.16 (1.41–3.30); *p* < 0.001	1.46 (0.97–2.20); *p* = 0.069
Marital status	1.60 (1.09–2.35); *p* = 0.017	1.10 (0.74–1.61); *p* = 0.645	1.08 (0.73–1.58); *p* = 0.704	1.65 (1.12–2.43); *p* = 0.011
Financial situation	1.07 (0.72–1.58); *p* = 0.733	3.27 (2.18–4.89); *p* < 0.001	1.79 (1.21–2.66); *p* = 0.004	0.93 (0.63–1.37); *p* = 0.706
Education	0.77 (0.52–1.12); *p* = 0.170	0.29 (0.20–0.43); *p* < 0.001	0.48 (0.33–0.71); *p* < 0.001	0.91 (0.62–1.33); *p* = 0.629
Leisure activity	1.20 (0.82–1.76); *p* = 0.347	0.46 (0.31–0.68); *p* < 0.001	0.64 (0.43–0.94); *p* = 0.022	1.38 (0.94–2.02); *p* = 0.097
Living arrangement	0.69 (0.46–1.01); *p* = 0.059	0.81 (0.55–1.19); *p* = 0.283	0.79 (0.54–1.17); *p* = 0.241	0.65 (0.44–0.95); *p* = 0.028
Gender	1.52 (1.00–2.31); *p* = 0.050	1.21 (0.80–1.84); *p* = 0.358	1.97 (1.30–3.00); *p* = 0.001	1.25 (0.82–1.88); *p* = 0.297
Frequency of meetings	1.18 (0.81–1.72); *p* = 0.383	1.26 (0.86–1.85); *p* = 0.227	1.63 (1.11–2.40); *p* = 0.012	1.40 (0.96–2.05); *p* = 0.079

*OR* odds ratio, *CI* confidence interval

Depressive symptoms were significantly associated with negative self-perception of ageing both for the overall AAQ score (OR = 2.07) and for all three AAQ domains. The strongest association was observed in the Physical Change domain (OR = 4.92), indicating that respondents with depressive symptoms had nearly fivefold higher odds of negative attitudes towards ageing in this domain compared with those without depressive symptoms. Loneliness was also significantly associated with higher odds of negative attitudes towards ageing, both for the overall AAQ score (OR = 2.59) and across all three AAQ domains. Poorer self-rated health was significantly associated with negative attitudes towards ageing for the overall AAQ score (OR = 2.07) as well as for each individual domain. Likewise, a poorly developed social network was associated with more negative attitudes towards ageing (OR = 1.72) and with all three AAQ domains. Family relationship quality rated as good or poorer was associated with more negative scores across the individual AAQ domains, although no significant association was observed for the overall AAQ score. The strongest association between poorer family relationship quality and negative attitudes towards ageing was found in the Physical Change domain (OR = 3.37). Age was also significantly associated with attitudes towards ageing. Older age was associated with higher odds of negative attitudes towards ageing both for the overall AAQ score (OR = 1.60) and for each AAQ domain. Poor financial situation was significantly associated with more negative attitudes only in the Physical Change domain (OR = 3.27).

For the remaining variables, some were associated with lower odds of negative attitudes towards ageing in individual AAQ domains as well as for the overall AAQ score (OR < 1). Higher educational attainment was significantly associated with lower odds of negative attitudes towards ageing in the Physical Change (OR = 0.29) and Psychological Growth (OR = 0.48) domains. Similarly, active leisure activities were associated with lower odds of negative attitudes in the Physical Change (OR = 0.46) and Psychological Growth (OR = 0.64) domains. Living with another person was associated with lower odds of negative attitudes towards ageing for the overall AAQ score (OR = 0.65). No significant associations were observed between sex, marital status, or frequency of meetings with significant others and most of the analysed AAQ domains; the observed associations were either marginal or did not reach statistical significance.

## Discussion

The aim of the present study was to examine the relationship between loneliness, depressive symptoms, and self-perception of ageing among older adults.

The first stage of the analysis assessed respondents’ attitudes towards their own ageing, both overall and across the three AAQ domains: Psychological Growth, Physical Change, and Psychosocial Loss. The findings are consistent with those reported in previous studies [6, 9]. The highest mean score was observed in the Psychological Growth domain, which reflects perceiving ageing as a period of continued personal development, gaining experience, and passing it on to others [21]. Studies conducted in Poland have shown that older adults often serve as authorities, sources of knowledge, and transmitters of life experience within their families and the wider community [26].

In contrast, the lowest scores were observed in the Psychosocial Loss domain. Negative perceptions in this domain reflect feelings of age-related exclusion, loneliness, and loss of social roles. Poorer self-rated health, unsatisfactory family relationships, and limited social contacts may intensify these experiences and contribute to loneliness, leading to reduced self-esteem and a diminished sense of social usefulness [27]. Negative perceptions of ageing may be further reinforced by the coexistence of age- and disability-related stereotypes. As highlighted in the literature, ageism and discrimination associated with functional limitations are closely intertwined. Older adults experiencing declining physical functioning are more likely to face social marginalisation, loss of autonomy, and reduced self-esteem, which may contribute to a more negative self-perception of ageing and lower engagement in social and health-promoting activities [4, 28].

Most respondents, however, demonstrated positive attitudes towards ageing in the Physical Change domain. This finding may partly reflect the characteristics of the study sample, which consisted of individuals who were able to participate in the study independently and therefore may have perceived their physical functioning more positively. Overall, the findings support the multidimensional nature of self-perception of ageing and indicate that the individual AAQ domains capture different aspects of the ageing experience that may be influenced by different determinants.

When interpreting the findings of the present study, the heterogeneity of the study sample with respect to participation in social activities should be taken into account. Additional analyses showed that participants attending Universities of the Third Age (UTA) were more likely to belong to the younger age group, to be married, to report a better financial situation, and to present fewer depressive symptoms than the remaining respondents. These characteristics have also been identified in previous studies as being associated with more positive attitudes towards ageing [6–8, 11]. This may partly explain the relatively high proportion of positive attitudes towards ageing observed in the present study.

In the next stage of the analysis, respondents were classified into two groups representing positive and negative attitudes towards ageing using cluster analysis. The findings demonstrated significant associations between negative self-perception of ageing and both depressive symptoms and loneliness. In addition, negative attitudes towards ageing were more common among older adults who lived alone, had a poorly developed social network, and reported poorer self-rated health.

Depressive symptoms appear to be closely associated with negative self-perception of ageing [11]. Pessimistic thinking and a negative interpretation of life experiences may lead individuals to focus predominantly on the adverse aspects of growing older. Chachamovich et al. demonstrated that even subclinical depressive symptoms were associated with a significant reduction in quality of life and more negative attitudes towards ageing among older adults [11]. Similarly, Silva et al. reported that more dysfunctional attitudes among older adults were associated with greater severity of depressive symptoms. The authors emphasised the importance of negative cognitive schemas and maladaptive beliefs in the psychological functioning of older adults [29]. These observations are consistent with our findings, in which depressive symptoms were significantly associated with more negative attitudes towards ageing both overall and across all AAQ domains.

It is noteworthy that the strength of these associations differed across the AAQ domains. The strongest association was observed in the Physical Change domain, where the presence of depressive symptoms was associated with nearly fivefold higher odds of negative attitudes towards ageing. This finding may suggest that older adults experiencing depressive symptoms are more likely to focus on deteriorating health, functional limitations, and declining physical capacity.

Previous studies have demonstrated associations between loneliness and depressive symptoms [30], as well as between loneliness and more negative attitudes towards ageing. Similar findings were reported by Silva et al. in a study of Portuguese older adults, in which higher levels of loneliness were associated with greater depressive symptom severity and poorer quality of life [31]. Likewise, Hu and Li demonstrated, in a longitudinal study of older Americans, that loneliness was associated with a more negative self-perception of ageing [32]. These findings are consistent with the results of the present study. Loneliness was significantly associated with negative attitudes towards ageing both overall and across all AAQ domains, with particularly strong associations observed in the Psychological Growth and Physical Change domains.

Our findings also showed that a poorly developed social network was associated with more negative self-perception of ageing both overall and across all AAQ domains. In addition, poorer family relationships were associated with more negative perceptions of ageing within the individual AAQ domains, particularly the Physical Change domain. This may reflect the important role of social support in maintaining activity and motivation to engage in health-promoting behaviours. Loneliness was also significantly associated with the Psychosocial Loss domain, which is consistent with previous studies showing that older adults with broader social networks and greater satisfaction with social support tend to report more positive attitudes towards their own ageing [12]. Conversely, the absence of social ties or deficiencies in social support may undermine the psychosocial functioning of older adults. The way older adults perceive their own ageing and the attitudes they adopt are largely influenced by family and social relationships, as well as by the level of support they receive from those around them [33].

Social participation is an important determinant of social functioning. According to Levy’s Stereotype Embodiment Theory, negative beliefs about ageing may influence older adults through psychological and behavioural mechanisms, leading, among other consequences, to reduced social participation, social withdrawal, and lower engagement in health-promoting behaviours [4, 20]. Conversely, greater social participation may foster a more positive self-perception of ageing [4]. Using data from the German Ageing Survey conducted in 2008 and 2014, Schwartz et al. examined the bidirectional relationship between social engagement and self-perception of ageing. Their findings suggest that social engagement may be an indicator of successful ageing. At the same time, more positive perceptions of ageing increase the likelihood of continued participation in social activities over time [34].

In the present study, active leisure participation was associated with more positive attitudes in the Physical Change and Psychological Growth domains, whereas the association was weaker in the Psychosocial Loss domain. Participation in Senior Day Care Clubs, Universities of the Third Age, or volunteer activities may represent an effective approach to reducing loneliness and increasing opportunities for social interaction among older adults.

Self-rated health is another important determinant of attitudes towards ageing. Previous studies have shown that health problems negatively influence perceptions of ageing, which older adults often associate with loss of vitality, dependence on others, and social exclusion. In a secondary analysis of survey data collected in 20 countries participating in the WHOQOL-OLD project, Low et al. demonstrated a significant association between self-rated health and attitudes towards ageing [2]. Respondents who rated their health as fair or poor reported more negative attitudes towards ageing both overall and across all three AAQ domains. Similarly, in the present study, poorer self-rated health was significantly associated with self-perception of ageing and with nearly twofold higher odds of negative attitudes towards ageing compared with respondents who rated their health positively.

Our findings also demonstrated a significant association between age and self-perception of ageing. These results are consistent with those reported in most previous studies examining this relationship [6, 35]. Perceptions of ageing may become increasingly negative with advancing age, potentially reflecting deteriorating health, progressive functional limitations, and the loss of significant others.

Educational attainment is another important factor that may facilitate adaptation to age-related changes. Studies examining the association between education and attitudes towards ageing consistently indicate that lower educational attainment is associated with more negative attitudes towards ageing [8]. These findings are consistent with the results of the present study, in which lower educational attainment was associated with more negative attitudes in the Physical Change and Psychological Growth domains. Higher educational attainment may facilitate better adaptation to later life and promote more effective coping with age-related changes.

### Strengths and limitations

A major strength of this study is the relatively large sample of 439 older adults and the use of validated research instruments. An additional strength is the multidimensional assessment of self-perception of ageing through the analysis of the three AAQ-24-PL domains: Psychosocial Loss, Physical Change, and Psychological Growth. The findings indicate that loneliness and depressive symptoms are not equally associated with all domains of self-perception of ageing, highlighting the importance of considering the multidimensional nature of attitudes towards ageing both in research and when planning interventions aimed at promoting well-being among older adults. Another strength of the study is the simultaneous examination of loneliness, depressive symptoms, and self-perception of ageing, an approach that remains relatively uncommon in the existing literature.

Several limitations should be considered when interpreting the findings. First, a convenience sampling approach was used, which may have introduced selection bias and limits the generalisability of the findings to the broader population of older adults. Second, only individuals able to participate independently in the study procedures were included. Consequently, the findings may not fully reflect the experiences of all older adults. In particular, individuals with poorer health, greater social isolation, or functional limitations may have been underrepresented, despite being at greater risk of loneliness and negative self-perception of ageing. Finally, the cross-sectional design enabled the identification of associations between the analysed variables but did not allow causal inferences to be drawn. Future research should consider probability sampling strategies and include older adults with varying levels of functional ability. Longitudinal studies are also warranted to clarify the direction and nature of the observed associations.

## Conclusions

Most older adults demonstrated positive attitudes towards their own ageing, particularly in the Physical Change and Psychological Growth domains, whereas more negative attitudes were observed in the Psychosocial Loss domain. Loneliness and depressive symptoms were the factors most strongly associated with negative self-perception of ageing. The findings also indicate that social relationships, self-rated health, and active leisure participation are associated with how older adults perceive their own ageing. These areas may represent important targets for interventions aimed at promoting well-being in later life.

## Data Availability

The datasets used and/or analysed during the current study are available from the corresponding author on reasonable request.

## Abbreviations

AAQ: Attitudes to Ageing Questionnaire
PL: Psychosocial Loss
PC: Physical Change
PG: Psychological Growth
GDS: 15–15–item Geriatric Depression Scale
DJGLS: De Jong Gierveld Loneliness Scale
AMTS: Abbreviated Mental Test Score
UTA: University of the Third Age
SDCC: Senior Day Care Club
